# Survival after allogeneic transplantation according to pretransplant minimal residual disease and conditioning intensity in patients with acute myeloid leukemia

**DOI:** 10.3389/fonc.2024.1394648

**Published:** 2024-05-02

**Authors:** Claudia Núñez-Torrón Stock, Carlos Jiménez Chillón, Fernando Martín Moro, Juan Marquet Palomanes, Miguel Piris Villaespesa, Ernesto Roldán Santiago, Eulalia Rodríguez Martín, Anabelle Chinea Rodríguez, Valentín García Gutiérrez, Gemma Moreno Jiménez, Javier López Jiménez, Pilar Herrera Puente

**Affiliations:** ^1^ Departamento de Hematología y Hemoterapia, Hospital Universitario Infanta Sofía, Madrid, Spain; ^2^ Medicine and Medical Specialties Department, Universidad Alcalá de Henares, Madrid, Spain; ^3^ Medicine Department, Universidad Europea de Madrid, Madrid, Spain; ^4^ Departamento de Hematología y Hemoterapia, Hospital Universitario Gregorio Marañón, Madrid, Spain; ^5^ Departamento de Hematología y Hemoterapia, Hospital Universitario Ramón y Cajal, Madrid, Spain; ^6^ Departamento de Inmunología, Hospital Universitario Ramón y Cajal, Madrid, Spain

**Keywords:** acute myeloid leukemia, allogeneic transplantation, minimal residual disease, conditioning intensity, monosomal karyotype

## Abstract

**Background:**

The measurement of minimal residual disease (MRD) by multiparametric flow cytometry (MFC) before hematopoietic stem cell transplantation (HSCT) in patients with acute myeloid leukemia (AML) is a powerful prognostic factor. The interaction of pretransplant MRD and the conditioning intensity has not yet been clarified.

**Objective:**

The aim of this study is to analyze the transplant outcomes of patients with AML who underwent HSCT in complete remission (CR), comparing patients with positive MRD (MRD+) and negative MRD (MRD−) before HSCT, and the interaction between conditioning intensity and pre-HSCT MRD.

**Study design:**

We retrospectively analyzed the transplant outcomes of 118 patients with AML who underwent HSCT in CR in a single institution, comparing patients with MRD+ and MRD− before HSCT using a cutoff of 0.1% on MFC, and the interaction between conditioning intensity and pre-HSCT MRD.

**Results:**

Patients with MRD+ before HSCT had a significantly worse 2-year (2y) event-free survival (EFS) (56.5% vs. 32.0%, *p* = 0.018) than MRD− patients, due to a higher cumulative incidence of relapse (CIR) at 2 years (49.0% vs. 18.0%, *p* = 0.002), with no differences in transplant-related mortality (TRM) (2y-TRM, 19.0% and 25.0%, respectively, *p* = 0.588). In the analysis stratified by conditioning intensity, in patients who received MAC, those with MRD− before HSCT had better EFS (*p* = 0.009) and overall survival (OS) (*p* = 0.070) due to lower CIR (*p* = 0.004) than MRD+ patients. On the other hand, the survival was similar in reduced intensity conditioning (RIC) patients regardless of the MRD status.

**Conclusions:**

Patients with MRD+ before HSCT have worse outcomes than MRD− patients. In patients who received MAC, MRD− patients have better EFS and OS due to lower CIR than MRD+ patients, probably because they represent a more chemo-sensitive group. However, among RIC patients, results were similar regardless of the MRD status.

## Introduction

Allogeneic hematopoietic stem cell transplantation (HSCT) is the only curative strategy for some patients with acute myeloid leukemia (AML) (1). A range of factors influence the outcomes after HSCT, but relapse is still the main cause of HCST failure. Determining factors may be related to the patient (2), AML biology [mostly cytogenetic and molecular alterations (3)], response to chemotherapy, and HSCT procedure (4, 5).

In terms of leukemia status prior to allograft, patients in complete remission (CR) have better outcomes than those transplanted with active disease (AD) (6, 7). Nowadays, different methods are available to measure minimal residual disease (MRD) in patients in CR, in which one of the most widely used is the multiparametric flow cytometry (MFC). Many reports showed that detectable MRD before HSCT has a negative impact on survival (5, 8, 9), determined mainly by a higher cumulative incidence of relapse (CIR).

The optimal conditioning intensity for patients in CR is not clear. Because prospective trials could not demonstrate a survival benefit for reduced intensity conditioning (RIC) over myeloablative conditioning (MAC) but the BMT CTN 0901 trial (10), the latter is still preferred for younger patients without comorbidities (11, 12). The interaction of conditioning intensity and pretransplant MRD also remains unresolved, and the results from previous published reports are contradictory (13, 14).

The aim of this study is to analyze the transplant outcomes of patients with AML who underwent HSCT in CR, comparing patients with positive MRD and negative MRD before HSCT, and the interaction between conditioning intensity and pre-HSCT MRD.

## Material and methods

The study protocol was reviewed and approved by the Ramón y Cajal Hospital Ethics Committee (243/21), and the study was conducted in accordance with the Declaration of Helsinki.

### Study population and variables

We performed a retrospective analysis of 118 consecutive adult patients (≥18 years) diagnosed with AML, who received HSCT between 1 January 2008 and 31 May 2021 at Ramón y Cajal Hospital in Madrid. All patients achieved CR with or without peripheral recovery before HSCT and had an assessable bone marrow sample performed using MFC at our center before HSCT. Bone marrow samples were obtained within 30 (± 15) days before the day of transplantation. We classified the cohort in two groups: patients with MRD <0.1% (MRD−) and patients with MRD ≥0.1% (MRD+) on pre-HSCT MFC. Disease-related variables included AML subtype according to the WHO 2016 classification (15), genetic risk according to the European LeukemiaNet 2017 risk classification (16), and the presence of complex karyotype and monosomal karyotype (MK) at diagnosis. Transplant-related variables included in the analysis were MRD status by MFC before HSCT, HCT-CI score, time from diagnosis to HSCT, conditioning intensity, conditioning scheme, graft-versus-host disease (GHVD) prophylaxis scheme, donor source, donor type, and median infused CD34+/kg.

### MRD assessment by MFC

Bone marrow samples from patients were obtained before HSCT. MRD assessment by MFC was performed as we previously described (17). Positive MRD was defined as ≥0.1% following the European LeukemiaNet (ELN) recommendations, as reported elsewhere (17, 18).

### Conditioning scheme and GHVD prophylaxis

Conditioning intensity was chosen according to the institutional strategy, taking into account the age and comorbidities of the patient at the time of HSCT. Although the MRD status before HSCT was known by the clinicians, it was not a factor influencing the choice of conditioning intensity, during the period of this study, and only patients transplanted with AD were proposed to intensify with sequential conditioning when possible. The conditioning scheme was cyclophosphamide + busulfan–based between 2008 and 2011 and, since 2011, fludarabine + busulfan–based. As previously reported, RIC was defined as a total busulfan dose of less than 9 mg/kg iv (19). GVHD prophylaxis comprised cyclosporine (CsA) and mycophenolate (MMF) for RIC; CsA and methotrexate for MAC; and post-transplant cyclophosphamide (PTCy), CsA, and MMF for haploidentical HSCT. Since 2019, we also used the post-transplant cyclophosphamide, CsA, and MMF strategy for patients with one or more HLA mismatches. Thymoglobulin and thiotepa were added in all patients with an unrelated donor. Thymoglobulin dose was 6 mg/kg iv for both MAC and RIC. For patients who received MAC, thiotepa dose was 10 mg/kg iv, and, in those cases, busulfan total dose was adjusted to 9.9 mg/kg iv. For patients who received RIC, thiotepa dose was 5mg/kg iv and busulfan dose was 6.4 mg/kg iv. Peripheral blood (PB) was the source of progenitor cells for all patients.

### Clinical endpoints and definitions

The primary endpoints of the study were CIR and event-free survival (EFS). Other endpoints of interest were overall survival (OS) and transplant-related mortality (TRM). We defined CR as <5% blasts on bone marrow cytology with no circulating blasts in PB and absence of extramedullary disease. OS was defined as the time from transplantation to death and EFS as the time from transplantation to either relapse or death. Relapse was considered as reappearance of ≥5% blasts in bone marrow, circulating blasts, or extramedullary disease. CIR was defined as time to onset of leukemia recurrence. TRM was defined as death without relapse (16, 20).

### Statistical analysis

The Chi-squared test or the Fisher’s test was used to compare differences between categorical variables, as well as the Student’s T-test or the Mann–Whitney U-test in the case of continuous variables. OS and EFS were estimated using the Kaplan–Meier method, and differences were analyzed using the log-rank test. CIR and non-relapse mortality were estimated using cumulative incidence method, and differences were estimated using the Gray’s method, considering each risk as a competing risk. All *p*-values were two-sided, and *p* < 0.05 was considered significant. Multivariate analyses were performed using Cox proportional hazards model for OS and EFS, and Fine–Gray proportional hazard regression was used for CIR including the variables MRD status (MRD− and MRD+) and the conditioning intensity (MAC and RIC) and adjusting for clinical and sociodemographic characteristics. SPSS v.22 (IBM) and XLSTAT 2020.5.1 software were used to perform the statistical analysis.

## Results

We analyzed 118 patients transplanted in CR with or without peripheral recovery who had available MRD data determined by MFC. Eighty-six patients (72.9%) were MRD−, and 32 patients (27.1%) were MRD+. The median follow-up of the overall cohort was 14 (6.75–51) months, and the median follow-up in MRD− and MRD+ groups was 12 (7–50) months and 17 (5.25–52.25) months, respectively. The median follow-up in survivors was 44 (11–69) months.

The baseline characteristics of each group and the overall cohort are reflected in **Table 1**. Baseline and transplant-related characteristics were balanced in both groups, except that MRD+ patients showed a higher rate of MKs (18.8% vs. 5.8%, *p* = 0.043) and a lower median number of infused CD34+ × 10^6^/kg (5.0 vs. 6.0, *p* = 0.009).

**Table 1:** Baseline characteristics in patients with MRD− and MRD+ and overall population.

Variables	MRD−, n = 86	MRD+, n = 32	Overall population, n = 118	P-value
**Male sex, *n* (%)**	48 (55.8%)	23 (71.9%)	71 (60.2%)	0.141
**Median age at HSCT, years (interquartile range)**	54 (45–60.25)	49.5 (35.5–61)	54 (42–61)	0.303
**Patients ≥ 60 years at HSCT, *n* (%)**	23 (26.7%)	10 (31.3%)	33 (28.0%)	0.649
WHO 2016 classification, *n* (%)				
AML with recurrent genetic abnormalities AML with myelodysplasia-related changes Therapy-related myeloid neoplasms AML, NOS Blastic phase of MPN Ph−	23 (26.7%) 24 (27.9%) 11 (12.8%) 26 (30.2%) 2 (2.3%)	9 (28.1%) 14 (43.8%) 4 (12.5%) 4 (12.5%) 1 (3.1%)	32 (27.1%) 38 (32.2%) 15 (12.7%) 30 (25.4%) 3 (2.5%)	0.311
ELN classification, *n* (%)				
Favorable risk Intermediate risk Adverse risk	17 (19.8%) 50 (58.1%) 19 (22.1%)	5 (15.6%) 16 (50.0%) 11 (34.4%)	22 (18.6%) 66 (55.9%) 30 (25.4%)	0.392
**Complex karyotype, *n* (%)**	8 (9.3%)	7 (21.8%	15 (12.7%)	0.121
**Monosomal karyotype, *n* (%)**	5 (5.8%)	6 (18.8%)	11 (9.3%)	0.043*
**Patients transplanted in first CR, *n* (%)**	72 (83.7%)	26 (81.3%)	98 (83.1%)	0.748
**Median time from diagnosis to HSCT, months (interquartile range)**	5 (4–7)	4 (3–7)	5 (4–7)	0.146
Conditioning intensity, *n* (%)				
Myeloablative Reduced intensity	51 (59.3%) 35 (40.7)	21 (65.6%) 11 (34.4%)	72 (61.0%) 46 (39.0%)	0.531
Conditioning regimen, *n* (%)				
FluBu BuCy TBF Cy-FluBu TBI-Cy FluMel	56 (65.1%) 3 (3.5%) 22 (25.6%) 4 (4.7%) 1 (1.2%) 0 (0.0%)	17 (53.1%) 4 (12.5%) 7 (21.9%) 3 (9.4%) 0 (0.0%) 1 (3.1%)	73 (61.9%) 7 (5.9%) 29 (24.6%) 7 (5.9%) 1 (0.8%) 1 (0.8%)	0.114
Donor type, *n* (%)				
Related donor Unrelated donor Haploidentical donor	37 (43.0%) 20 (23.3%) 29 (33.7%)	14 (43.8%) 8 (25.0%) 10 (31.2%)	51 (43.2%) 28 (23.7%) 39 (33.1%)	0.963
Donor source, *n* (%)				
Peripheral blood Bone marrow	85 (98.8%) 1 (1.2%)	100% (100.0%) 0 (0.0%)	117 (99.2%) 1 (0.8%)	1.000
GVHD prophylaxis scheme, *n* (%)				
**PTCy + MMF + CsA** **MMF + CsA** **MTX + CsA**	33 (38.4%) 19 (22.1%) 34(39.5%)	11 (34.4%) 4 (12.5%) 17 (53.1%)	44 (37.3%) 23 (19.5%) 51 (43.2%)	0.597
HCT-CI score, *n* (%)				
0 1–2 ≥3 Missing	44 (51.2%) 16 (18.6%) 18 (20.9%) 8 (9.3%)	17 (53.1%) 6 (18.8%) 5 (15.6%) 4 (12.5%)	61 (51.7%) 22 (18.6%) 23 (19.5%) 12 (10.2%)	0.846
**CD34 + ^6^/kg, median (range)**	6.0 (4.3–6.29)	5.0 (4.0–5.33)	5.3 (4.2–6.1)	0.009*

*p-value < 0.05; BuCy, busulfan + cyclophosphamide; CR, complete remission; Cy-FluBu, cyclophosphamide + fludarabine + busulfan; ELN, European LeukemiaNet; FluBu, fludarabine + busulfan; FluMel, fludarabine + melphalan; GVHD, graft-versus-host disease; HCT-CI, hematopoietic cell transplantation–specific comorbidity index; HSCT, hematopoietic stem cell transplantation; MMF + CsA, mycophenolate + cyclosporine; MPN Ph−, myeloproliferative neoplasm Philadelphia negative; MRD, minimal residual disease; MTX + CsA, methotrexate + cyclosporine; NOS, not otherwise specified; PTCy + MMF + CsA, post-HSCT cyclophosphamide; TBF, thiotepa + busulfan + fludarabine; TBI-Cy, total body irradiation + cyclophosphamide; WHO, World Health Organization.

Ninety-nine patients received intensive chemotherapy before HSCT, with a median of three cycles before HSCT (2–4). Sixteen patients received intensive chemotherapy and then any cycle with hypomethylating agents before HSCT, one patient received intensive chemotherapy and then six cycles of azacitidine + venetoclax, and two patients only received hypomethylating agents before HSCT.

Regarding the conditioning intensity, 70 of the 118 (59.3%) patients received MAC, and 48/118 (40.7%) patients received RIC. Seven of the 118 patients received cyclophosphamide + busulfan, and 109 of the 118 patients received fludarabine + busulfan–based regimens. One patient received fludarabine + melphalan, and one patient received cyclophosphamide + total body irradiation by decision of the HSCT team. Among patients receiving MAC, 19 (27.1%) patients had MRD+ before HSCT compared to 13 (27.1%) patients who received RIC (*p* = 0.994). More patients receiving RIC than MAC had an HCT-CI score ≥3 (36.2% vs. 10.2%, *p* = 0.005), and they were older (among patients who received RIC, 64.6% were ≥ 60 years old vs. 2.9% among those who received MAC, *p* < 0.001).

### Overall survival and event-free survival

**Figure 1:**
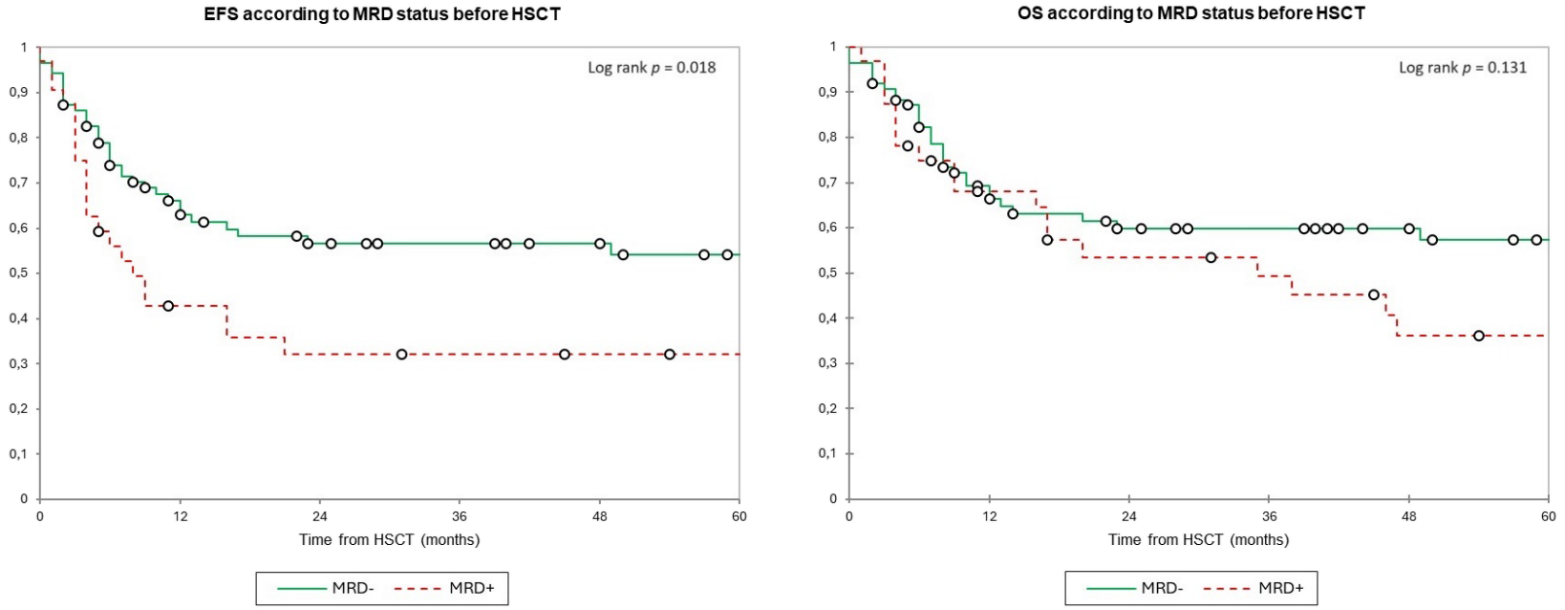
Event-free survival and overall survival according to minimal MRD status by MFC before HSCT. Estimates of (left) EFS and (right) OS after HSCT for patients with AML in complete remission according to the MRD status, shown individually for MRD− (n = 86) and MRD+ (n = 32), respectively. Patients with MRD− have significantly worse EFS (2y-EFS 56.5% vs. 32.0%) with no significant differences for OS (2y-OS was 60.0% vs. 53.5%). EFS, event-free survival; HSCT, hematopoietic stem cell transplantation; MRD, minimal residual disease; OS, overall survival.

**Figure 2:**
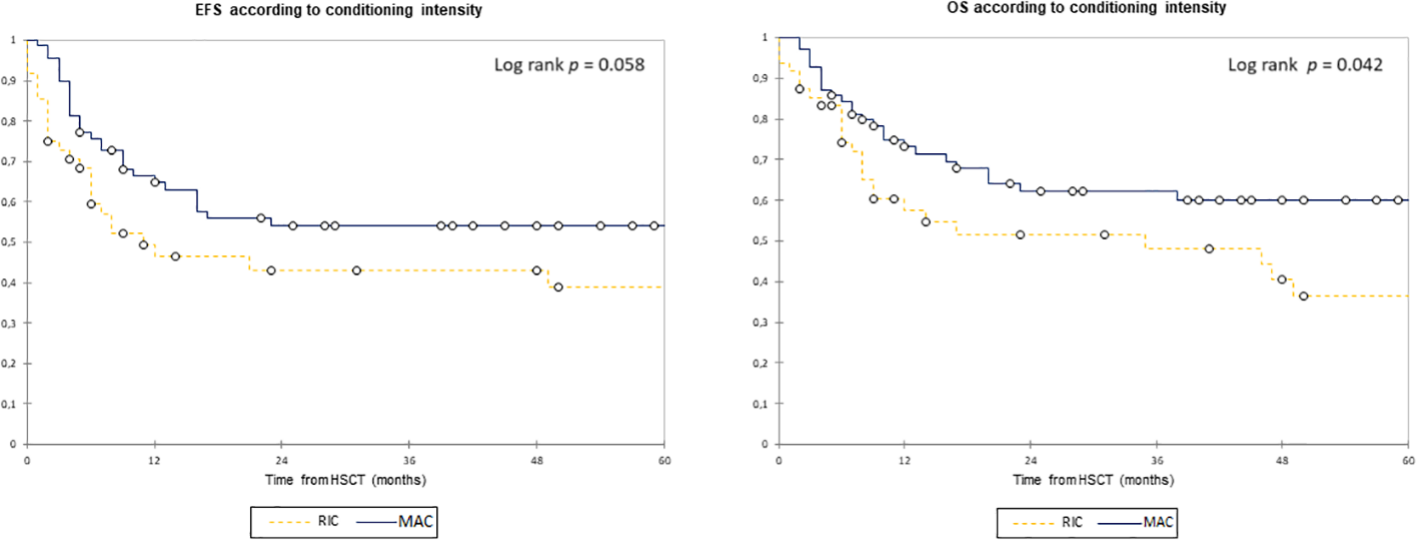
Event-free survival and overall survival according to conditioning intensity. Estimates of (left) EFS and (right) OS after HSCT for patients with AML in complete remission according to conditioning intensity, shown individually for MAC (n = 70) and RIC (n = 48), respectively. Patients with MAC− have a trend toward better EFS (2y-EFS, 54.0% vs. 43.0%) and significantly better OS (62.0% vs. 51.5%). EFS, event-free survival; HSCT, hematopoietic stem cell transplantation; MAC, myeloablative conditioning; OS, overall survival; RIC, reduced intensity conditioning.

The 2-year EFS (2y-EFS) in the overall cohort was 49.5% with a 2y-OS of 58.0%. The 2y-EFS in patients with MRD− was 56.5% vs. 32.0% in the MRD+ group (*p* = 0.018), whereas the 2y-OS was 60.0% vs. 53.5% (*p* = 0.131), respectively (**Figure 1**). The 2y-EFS in patients who received MAC was 54.0% vs. 43.0% in those who received RIC (*p* = 0.058), whereas the 2y-OS was 62.0% vs. 51.5% (*p* = 0.042), respectively (**Figure 2**).

**Figure 3:**
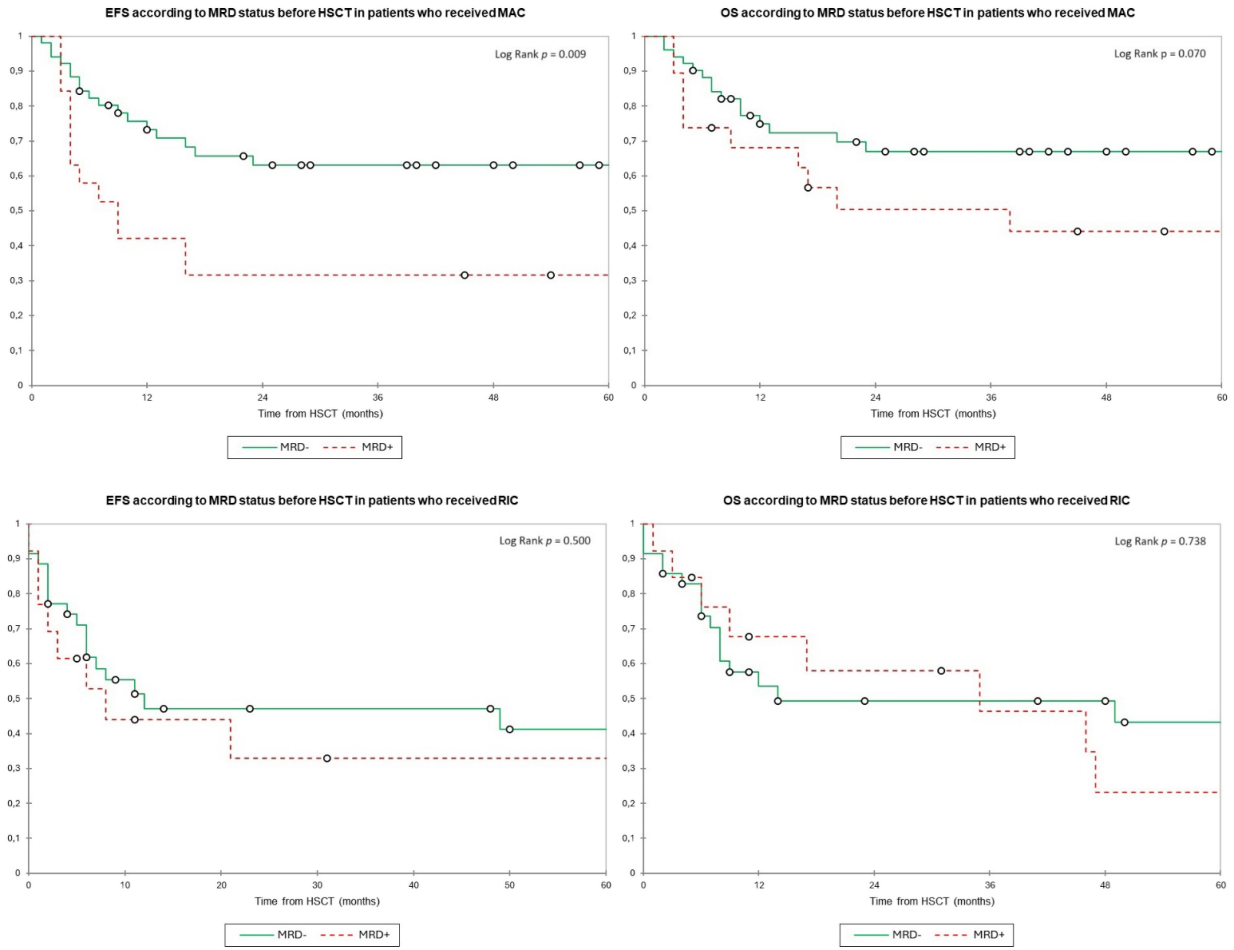
Event-free survival and overall survival according to the MRD status before HSCT and stratified by conditioning intensity. Estimates of (upper left) EFS and (upper right) OS after HSCT for patients with AML in complete remission according to the MRD status before HSCT among patients who received MAC, shown individually for MRD− (n = 51) and MRD+ (n = 19), respectively. Patients with MRD− have significantly better EFS (2y-EFS, 63.0% in MRD− vs. 31.5.0% in MRD+) and a trend to better OS (2y-OS, 67.0% vs. 50.5%) than patients with MRD+. Estimates of (lower left) EFS and (lower right) OS after HSCT for patients with AML in complete remission according to the MRD status before HSCT among patients who received RIC, shown individually for MRD− (n = 35) and MRD+ (n = 13), respectively. Patients have similar EFS (2y-EFS, 47.0% for MRD− vs. 33.0% for RIC) and OS (2y-OS, 49.0% vs. 58.0%) regardless of conditioning intensity. EFS, event-free survival; HSCT, hematopoietic stem cell transplantation; MRD, minimal residual disease; MAC, myeloablative conditioning; OS, overall survival; RIC, reduced intensity conditioning.

Patients were stratified according to conditioning intensity to analyze the specific impact of MRD status before transplant on each group. For MAC patients, the 2y-EFS was 63.0% in those with MRD− vs. 31.5% for MRD+ patients (*p* = 0.009), and the 2y-OS was 67.0% vs. 50.5% (*p* = 0.070), respectively. For patients who received RIC, the 2y-EFS was 47.0% vs. 33.0% (*p* = 0.500), and the 2y-OS was 49.0% vs. 58.0% (*p* = 0.738) (**Figure 3**).

We performed a univariate analysis and a multivariate analysis for both EFS and OS. A positive MRD before transplant [hazard ratio (HR), 1.92 (1.01–3.62)] was associated with worse EFS. Receiving a RIC was statistically significant for univariate analysis for EFS and OS but not in the multivariate analysis [HR, 1.80 (0.94–3.45) for EFS; HR, 1.09 (0.52–2.28) for OS]. A history of MPN Ph− in blastic phase [HR, 5.87 (1.19–29.04)] and Sorror score ≥3 [HR, 2.29 (1.13–4.05)] also had a negative impact on EFS. Patients older than 60 at HSCT [HR, 2.14 (1.13–4.05)] had a significantly worse OS. The presence of an MK at diagnosis was statistically significant for both EFS [HR, 3.97 (1.80–8.79)] and OS [HR, 4.20 (2.02–8.74)] (**Tables 2**, **3**).

**Table 2:** Univariate and multivariate analysis for event-free survival.

Variable	Univariate analysis		Multivariate analysis&	
	Hazard ratio (CI 95%)	*p*-value	Hazard ratio (CI 95%)	*p*-value
WHO 2016 classification				
Recurrent genetic abnormalities (ref)				
Myelodysplasia-related changes	**2.56 (1.25–5.26)**	0.010*	2.16 (0.84–5.57)	0.113
Therapy-related	**3.08 (1.31–7.27)**	0.010*	2.83 (1.78–10.328)	0.115
Not otherwise specified	1.09 (0.45–2.63)	0.853		
Blastic phase of MPN Ph−	3.77 (0.83–17.06)	0.085	**5.87 (1.19–29.04)**	0.030*
ELN 2017 risk classification#				
Favorable risk (ref)				
Intermediate risk	1.79 (0.75–4.31)	0.191		
Adverse risk	**3.01 (1.21–7.49)**	0.018*		
Complex karyotype#				
No (ref)				
Yes	**3.03 (1.55–5.90)**	0.001*		
Monosomal karyotype				
No (ref)				
Yes	**3.94 (1.88–8.25)**	< 0.001*	**3.97 (1.80–8.78)**	0.001*
MRD status by MFC before HSCT				
Negative (<0.1%) (ref)				
Positive (≥0.1)	**1.88 (1.10–3.24)**	0.022*	**1.92(1.01–3.62)**	0.046*
Complete remission before HSCT				
CR1 (ref)				
≥CR2	1.13 (0.57–2.25)	0.718		
HCT-CI score at HSCT				
<3 (ref)				
≥3	**1.91 (1.04–3.50)**	0.037*	**2.29 (1.17–4.50)**	0.016*
Conditioning intensity				
Myeloablative (ref)				
Reduced Intensity	**1.78 (1.05–3.01)**	0.032*	1.8 (0.94–3.45)	0.077
Age at HSCT				
<60 years (ref)				
≥60 years	1.52 (0.87–2.67)	0.145		

*p-value < 0.05; CR1, first complete remission; CR2, second complete remission; ELN, European LeukemiaNet; HCT-CI hematopoietic cell transplantation–specific comorbidity index. HSCT, hematopoietic stem cell transplantation; MRD, minimal residual disease; MFC, multiparameter flow cytometry; MPN Ph−, myeloproliferative neoplasm Philadelphia negative; NOS, not otherwise specified; WHO, World Health Organization.

&Variables with a p-value < 0.100 and those that are confounding were included.

Variables were not included in the multivariate analysis due to high collinearity with the monosomic karyotype variable.

All values in bold correspond to those marked with an *, as specified in the legend of each table, indicating p < 0.05.

**Table 3:** Univariate and multivariate analysis for overall survival.

Variable	Univariate analysis		Multivariate analysis&	
	Hazard ratio (CI 95%)	*p*-value	Hazard ratio (CI 95%)	*p*-value
WHO 2016 classification				
Recurrent genetic abnormalities (ref)				
Myelodysplasia-related changes	**2.74 (1.29–5.83)**	0.009*	2.045 (0.84–5.00)	0.116
Therapy-related	**2.58 (1.04–6.37)**	0.040*	2.00 (0.67–5.92)	0.213
Not otherwise specified	1.10 (0.43–2.80)	0.838		
Blastic phase of MPN Ph−	3.42 (0.73–15.95)	0.12		
ELN 2017 risk classification#				
Favorable risk(ref)				
Intermediate risk	**1.68 (0.69–4.07)**	0.253*	2.11 (0.72–6.168)	0.173
Adverse risk	**2.45 (0.97–6.18)**	0.058*	2.29 (0.67–7.83)	0.188
Complex karyotype#				
No (ref)				
Yes	**2.49 (1.46—5.58)**	0.002*		
Monosomal karyotype				
No (ref)				
Yes	**3.94 (1.91–8.15)**	< 0.001*	**4.196 (2.02–8.74)**	< 0.001*
MRD status by MFC before HSCT				
Negative (<0.1%) (ref)				
Positive (≥0.1)	1.54 (0.87–2.72)	0.138		
Complete remission before HSCT				
CR1 (ref)				
≥CR2	1.34 (0.67–2.68)	0.411		
HCT-CI score at HSCT				
<3 (ref)				
≥3	**1.85 (0.98–3.48)**	0.059*	1.14 (0.44–2.94)	0.772
Conditioning intensity				
Myeloablative (red)				
Reduced intensity	**1.86 (1.07–3.23)**	0.028*	1.09 (0.52–2.28)	0.826
Age at HSCT				
<60 years (ref)				
≥60 years	**2.01 (1.13–3.59)**	0.018*	**2.14 (1.13–4.05)**	0.019*

*p-value < 0.05; CR1, first complete remission; CR2, second complete remission; ELN, European LeukemiaNet; HCT-CI, hematopoietic cell transplantation–specific comorbidity index. HSCT, hematopoietic stem cell transplantation; MPN Ph−, myeloproliferative neoplasm Philadelphia negative; MRD, minimal residual disease; MFC, multiparameter flow cytometry; NOS, not otherwise specified; WHO, World Health Organization.

&Variables with a p-value < 0.100 and those that are confounding were included.

Variables were not included in the multivariate analysis due to high collinearity with the monosomic karyotype variable.

All values in bold correspond to those marked with an *, as specified in the legend of each table, indicating p < 0.05.

### Transplant-related mortality and cumulative incidence of relapse

In the overall cohort, 30 patients relapsed after HSCT (15/86 MRD− and 15/32 MRD+ patients). The median time from HSCT to relapse was 5 (3–11.25) months. Twenty-five patients died after relapse during study follow-up, with a median time since relapse to death of 3 (1.75–14) months.

Twenty-six patients died without evidence of relapse (20/86 MRD− and 6/32 MRD+ patients). The median time from HSCT to TRM was 4 (2–8.25) months. The main cause of death was infectious complications in 14 patients, GVHD in two, mixed (infectious complication and GVHD) in five, cerebral edema in one, veno-occlusive disease in one, and fatal hemorrhage in another. The cause of death was unknown in two patients.

**Figure 4:**
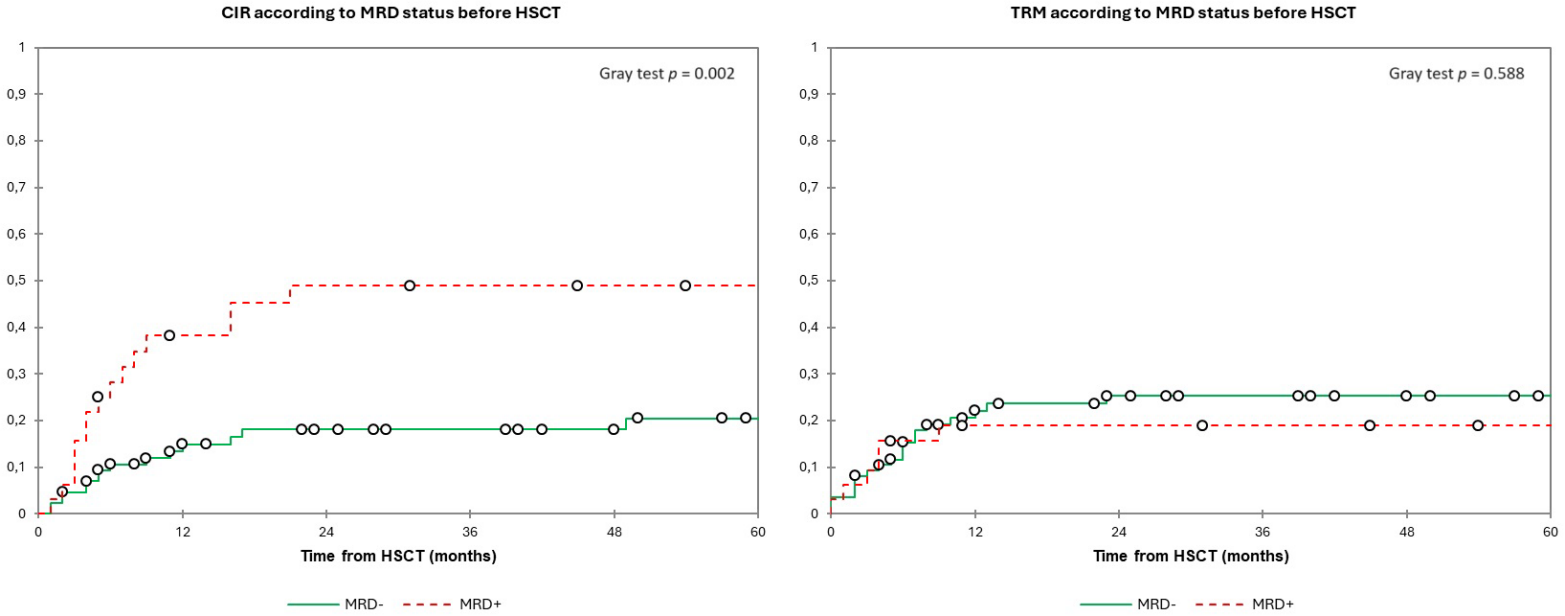
Estimated cumulative incidence of relapse and transplant-related mortality according to the MRD status before HSCT. Estimates of (left) CIR and (right) TRM after HSCT for patients with AML in complete remission according to the MRD status, shown individually for MRD− (n = 86) and MRD+ (n = 32), respectively. Patients with MRD+ had a significantly higher 2y-CIR (49.0%) compared to 18.0% in MRD− patients with no differences in 2y-TRM (19.0% and 25.0%, respectively). CIR, cumulative incidence of relapse; HSCT, hematopoietic stem cell transplantation; MRD, minimal residual disease; TRM, transplant-related mortality.

**Figure 5:**
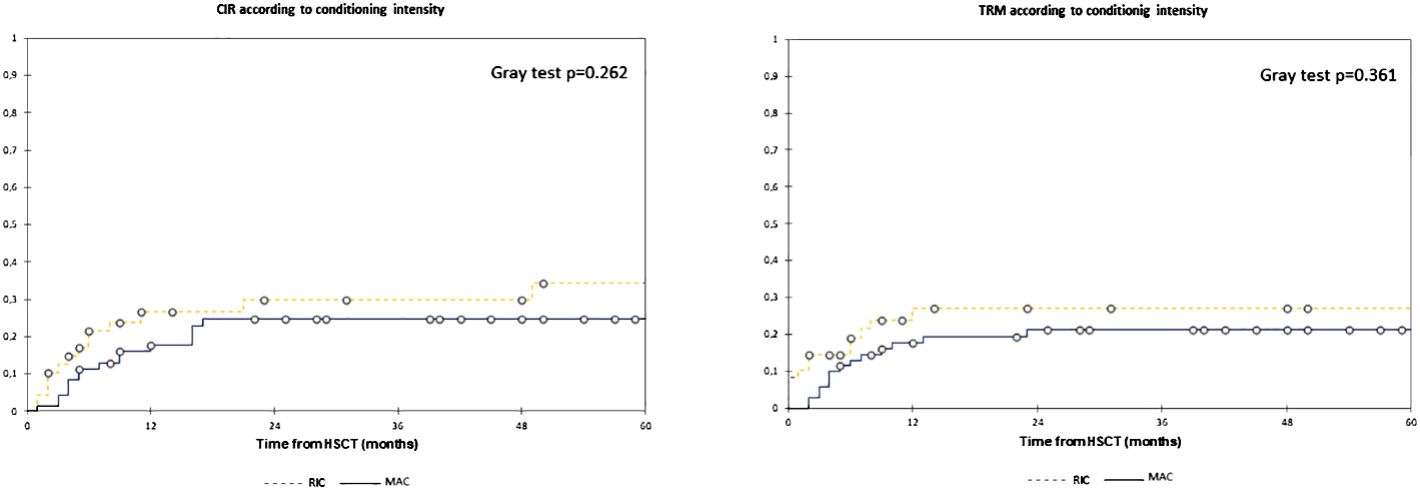
Estimated cumulative incidence of relapse and transplant-related mortality according to conditioning intensity. Estimates of (left) EFS and (right) OS after HSCT for patients with AML in complete remission according to conditioning intensity, shown individually for MAC (n = 70) and RIC (n = 48), respectively. Patients have similar CIR (2y-CIR, 24.5% for MAC and 30.0% for RIC) and TRM (2y-TRM, 21.0% vs. 27.0%) regardless of conditioning intensity. CIR, cumulative incidence of relapse; HSCT, hematopoietic stem cell transplantation; MAC, myeloablative conditioning; RIC, reduced intensity conditioning; TRM, transplant-related mortality.

**Figure 6:**
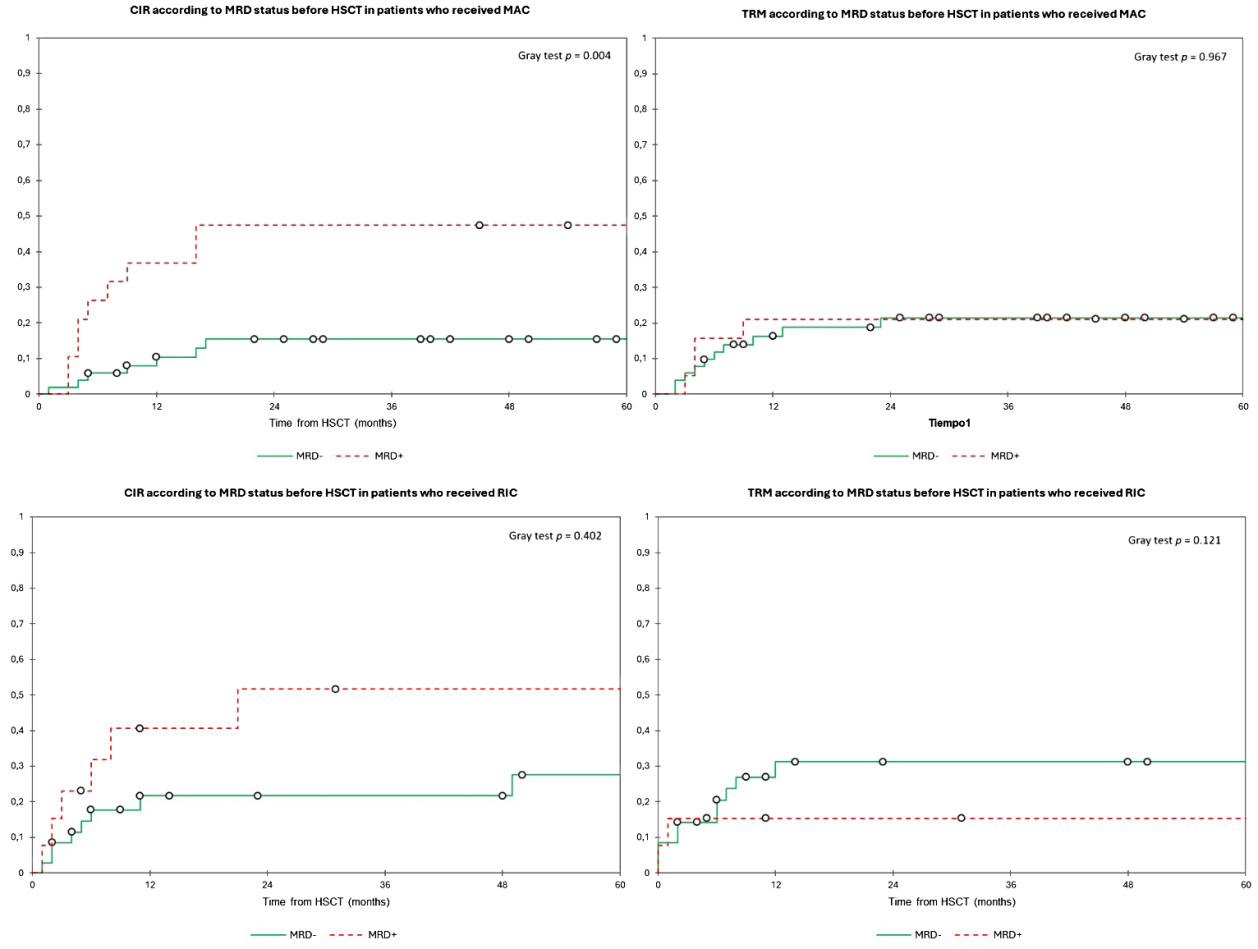
Estimated cumulative incidence of relapse and transplant-related mortality according to the MRD status before HSCT and stratified by conditioning intensity. Estimates of (upper left) CIR and (upper right) TRM after HSCT for patients with AML in complete remission according to the MRD status before HSCT among patients who received MAC, shown individually for MRD− (n = 51) and MRD+ (n = 19), respectively. Patients with MRD− have significantly lower CIR (2y-CIR, 15.0% in MRD− vs. 47.5% in MRD+) with no differences in TRM (2y-TRM, 21.5% vs. 21.0%) compared to MRD+. Estimates of (lower left) CIR and (lower right) TRM after HSCT for patients with AML in complete remission according to the MRD status before HSCT among patients who received RIC, shown individually for MRD− (n = 35) and MRD+ (n = 13), respectively. Patients had no statistically significant differences for both CIR (2y-CIR, 21.5% for MRD− vs. 52.0% for RIC) and TRM (2y-TRM, 31.0% vs. 15.0%). CIR, cumulative incidence of relapse; TRM, transplant-related mortality; HSCT, hematopoietic stem cell transplantation; MRD, minimal residual disease; MAC, myeloablative conditioning; OS, overall survival; RIC, reduced intensity conditioning.

In the overall population, the 2y-CIR was 26.5%, and the 2y-TRM was 23.5%. Patients with MRD+ had a significantly higher 2y-CIR (49.0%) compared to 18.0% in MRD− patients (*p* = 0.002) with no differences in 2y-TRM (19.0% and 25.0%, respectively, *p* = 0.588) (**Figure 4**). We found no differences in relapse incidence irrespective of conditioning intensity, with a 2y-CIR of 24.5% for MAC and 30.0% for RIC (*p* = 0.262), and a 2y-TRM of 21.0% vs. 27.0%, respectively (*p* = 0.361) (**Figure 5**). For MAC patients, the 2y-CIR was 15.0% in those with MRD− vs. 47.5% for MRD+ patients (*p* = 0.004), and the 2y-TRM was 21.5% vs. 21% (*p* = 0.967), respectively. For patients who received RIC, the 2y-CIR was 21.5% in MRD− vs. 52.0% in MRD+ (*p* = 0.402), and the 2y-TRM was 31.0% vs. 15.0%, respectively (*p* = 0.121) (**Figure 6**). In the multivariate analysis for CIR, MK [HR, 4.438 (1.77–11.14)] and MRD [HR, 2.74 (1.24–6.02)] were significantly associated with an increased risk of relapse after HSCT (**Table 4**).

**Table 4:** Univariate and multivariate analyses for cumulative incidence of relapse.

Variable	Univariate analysis		Multivariate analysis&	
	Hazard ratio (CI 95%)	P-value	Hazard ratio (CI 95%)	P-value
WHO 2016 classification				
Recurrent genetic abnormalities (ref)				
Myelodysplasia-related changes	1.51 (0.64–3.54)	0.346		
Therapy-related	2.30 (0.79–6.68)	0.126		
Not otherwise specified	0.46 (0.12–1.69)	0.241		
Blastic phase of MPN Ph−	1.66 (0.16–16.89)	0.668		
ELN 2017 risk classification#				
Favorable risk(ref)				
Intermediate risk	1.16 (0.31–4.30)	0.821		
Adverse risk	**4.71 (1.32–16.87)**	0.017*		
Complex karyotype#				
No (ref)				
Yes	**4.69 (2.04–10.76)**	<0.001*		
Monosomal karyotype				
No (ref)				
Yes	**5.99 (2.59–13.87)**	<0.001*	**4.44 (1.77–11.14)**	0.001*
MRD status by MFC before HSCT				
Negative (<0.1%) (ref)				
Positive (≥0.1)	**3.08 (1.52–6.24)**	**0.002**	**2.74 (1.24–6.02)**	0.012*
Complete remission before HSCT				
CR1 (ref)				
≥CR2	0.34 (0.08–1.43)	0.142		
HCT-CI score at HSCT				
<3 (ref)				
≥3	1.35 (0.58–3.14)	0.492		
Conditioning intensity				
Myeloablative (red)				
Reduced Intensity	1.76 (0.87–3.56)	0.113		
Age at HSCT				
<60 years (ref)				
≥60 years	0.87 (0.39–1.98)	0.744		

*p-value < 0.05; CR1, first complete remission; CR2, second complete remission; ELN, European LeukemiaNet; HCT-CI, hematopoietic cell transplantation–specific comorbidity index, HSCT, hematopoietic stem cell transplantation; MFC, multiparameter flow cytometry; MPN Ph−, myeloproliferative neoplasm Philadelphia negative; MRD, minimal residual disease; NOS, not otherwise specified; WHO, World Health Organization.

&Variables with a p-value < 0.100 and those that are confounding were included.

Variables were not included in the multivariate analysis due to high collinearity with the monosomic karyotype variable.

All values in bold correspond to those marked with an *, as specified in the legend of each table, indicating p < 0.05.

## Discussion

In our cohort, patients with MRD+ before HSCT by MFC had a significantly worse 2y-EFS due to a higher incidence of relapse with similar TRM than those who were MRD−. Several other published studies also reported that MRD+ had an impact on EFS due to an increased CIR (5, 17, 21, 22). Furthermore, in many others, MRD+ also had an impact on OS (8, 13, 23–34). Although the incidence of relapse was higher in MRD+ patients, that did not have a statistically significant impact on OS in our cohort, maybe due to the shortest follow-up in the MRD− group. An interesting data of our cohort is the different impact of MRD status according to conditioning intensity. Whereas in patients who received MAC, achieving MRD− before HSCT translated in better EFS and OS due to lower relapse incidence, survival in RIC patients was similar regardless of the MRD status. Although the retrospective nature does not allow to make direct comparisons between MAC and RIC populations due to baselines differences, MAC could not abrogate the adverse prognosis of MRD+ before HSCT, and the survival of MRD+ patients who received MAC was similar to RIC patients (both MRD− and MRD+). In line with our results, several studies demonstrate significant differences depending on the MRD status in patients receiving MAC, whereas this factor did not influence on OS and EFS on RIC patients (23, 28, 32, 34). In the meta-analysis performed by Buckley et al., patients with MRD+ by different methods, including several studies using MFC, had significantly worse EFS an OS, and MAC was unable to attenuate the negative effects of a positive pretransplant MRD. Moreover, the HRs for the impact of MRD were higher for studies in which >75% of patients received MAC than in those that included exclusively RIC or nonmyeloablative conditioning, although the wide confidence intervals did not allow definitive conclusions (8).

Araki et al. in a retrospective MAC cohort including patients in CR and patients transplanted with AD showed significant differences in survival even using any level of detectable MRD as positive by MFC, with an incidence of relapse and survival in MRD+ patients similar to patients with AD before HSCT (23). The differences on survival in patients who receive MAC according to MRD as we are reporting could be explained because of a higher chemosensitivity of MRD− patients compared to that of MRD+ patients. In our report, the survival differences in MAC patients according to MRD were due to an increased CIR in MRD+ patients. On the other hand, the key in MRD+ patients maybe is not to intensify conditioning intensity as is reasonable that they represent a group of chemo-resistant AMLs. For these patients who are at high risk of relapse, a possible strategy may be to focus on pretransplant MRD eradication and post-HSCT strategies to improve the graft-versus-leukemia (GVL) phenomenon and target and non-target maintenance therapies (35). On the other hand, some studies have failed to find differences in the interaction of pre-HSCT MRD and conditioning intensity, and some have even found a greater benefit of MAC for MRD+ patients, with similar results compare to MRD− with this conditioning intensity (4, 5, 14, 21, 31, 36). Hourigan et al. (4) conducted a study with pre-HSCT PB samples from patients included in the BMT CTN 0901 phase III trial. Whereas the results in MRD− patients determined by next-generation sequencing (NGS) were similar regardless of conditioning intensity, MRD+ patients who received RIC experienced higher rates of relapse and lower survival rates compared with those who received MAC (10). Therefore, in contrast to our report, some authors suggest that MAC has a greater benefit in patients with MRD+ and that RIC may be sufficient for the MRD− group to avoid unnecessary myelotoxicity (37).

In our cohort, 30 patients relapsed during the post-HSCT follow-up, mainly during first year after HSCT (76.7%, data not shown), as previously reported (38). The prognosis after post-HSCT relapse was very adverse with a very short time from relapse to death, which implies a narrow window for intervention. Therefore, we must continue to work on pre-emptive strategies in both pre- and post-HSCT periods in patients at higher risk of post-HSCT relapse, such as those with MRD+ prior to transplantation.

Other relevant prognostic factors that emerged in our study included the finding in the multivariate analysis that patients with MK had especially poor prognosis, with worse EFS and OS due to a higher CIR. In line with our results, this group of patients presented adverse survival in previous studies, even among transplanted patients (39). Another study by Morsink et al. (26) that analyzed the impact of MRD in patients with and without MK showed that, although patients with MK had worse survival, they did benefit from achieving pre-HSCT MRD−. In contrast to our findings, this group reported that having an MK was not independently associated with worse outcomes in the multivariate analysis. Because of the small number of patients with MK in our study, we were unable to analyze the impact of MRD in this subgroup.

The main limitations of this report are those inherent to the retrospective nature of our study. One of our strengths is that all patients were transplanted in the same center, which provides homogeneity in the conditioning scheme and the MRD measurement compared to previous multicenter studies (8, 24, 36). The intensity of conditioning was based on age and comorbidities as agreed by our transplant committee and not according to the level of MRD. Our study may shed light on whether MRD− patients who are suitable still benefit more from MAC because they are likely to present greater sensitivity to chemotherapy and, consequently, have lower CIR without significant increased TRM.

In conclusion, MRD+ before HSCT determined by MFC is an adverse prognostic factor and has an impact on EFS due to a higher risk of relapse in transplanted AML patients. The MRD status is especially relevant in patients who receive MAC, in whom MRD− before HSCT translates in better EFS and OS due to lower relapse incidence than MRD+. On the other hand, the survival was similar in RIC patients regardless of the MRD status. Whereas MRD− patients benefit more than MRD+ from receiving MAC when possible, the survival in MRD+ patients was adverse in both MAC and RIC populations. Future prospective trial could help us to clarify whether MRD+ patients benefit more from pre-HSCT treatments to eradicate MRD or post-HSCT strategies focused on improving GVL and maintenance therapies rather than on intensifying the conditioning intensity.

## Data availability statement

The raw data supporting the conclusions of this article will be made available by the authors, without undue reservation.

## Ethics statement

The study protocol was reviewed and approved by the Ramón y Cajal Hospital Ethics Committee (243/21). The studies were conducted in accordance with the local legislation and institutional requirements. Written informed consent for participation was not required from the participants or the participants’ legal guardians/next of kin in accordance with the national legislation and institutional requirements.

## Author contributions

CN-TS: Conceptualization, Investigation, Methodology, Supervision, Validation, Visualization, Writing – original draft, Writing – review & editing. CJC: Writing – original draft, Writing – review & editing. FMM: Writing – review & editing. JMP: Writing – review & editing. MPV: Writing – review & editing. ERS: Writing – review & editing. ERM: Writing – review & editing. ACR: Writing – review & editing. VGG: Writing – review & editing. GMJ: Writing – review & editing. JLJ: Writing – review & editing. PHP: Visualization, Writing – original draft, Writing – review & editing.
